# The Oxidized Protein Repair Enzymes Methionine Sulfoxide Reductases and Their Roles in Protecting against Oxidative Stress, in Ageing and in Regulating Protein Function

**DOI:** 10.3390/antiox7120191

**Published:** 2018-12-12

**Authors:** Sofia Lourenço dos Santos, Isabelle Petropoulos, Bertrand Friguet

**Affiliations:** Sorbonne Université, CNRS, INSERM, Institut de Biologie Paris-Seine, Biological Adaptation and Ageing, B2A-IBPS, F-75005 Paris, France; sofia.lourenco.santos@gmail.com

**Keywords:** protein oxidation, methionine oxidation, methionine sulfoxide reductases, oxidized protein repair, ageing

## Abstract

Cysteine and methionine residues are the amino acids most sensitive to oxidation by reactive oxygen species. However, in contrast to other amino acids, certain cysteine and methionine oxidation products can be reduced within proteins by dedicated enzymatic repair systems. Oxidation of cysteine first results in either the formation of a disulfide bridge or a sulfenic acid. Sulfenic acid can be converted to disulfide or sulfenamide or further oxidized to sulfinic acid. Disulfide can be easily reversed by different enzymatic systems such as the thioredoxin/thioredoxin reductase and the glutaredoxin/glutathione/glutathione reductase systems. Methionine side chains can also be oxidized by reactive oxygen species. Methionine oxidation, by the addition of an extra oxygen atom, leads to the generation of methionine sulfoxide. Enzymatically catalyzed reduction of methionine sulfoxide is achieved by either methionine sulfoxide reductase A or methionine sulfoxide reductase B, also referred as to the methionine sulfoxide reductases system. This oxidized protein repair system is further described in this review article in terms of its discovery and biologically relevant characteristics, and its important physiological roles in protecting against oxidative stress, in ageing and in regulating protein function.

## 1. Introduction

Enzymatically repair of protein oxidative damage is only possible for certain oxidation products of the sulfur-containing amino acids, cysteine and methionine. In the case of cysteine, the major systems involved in reversing the oxidation of disulfide bridges and sulfenic acid include the reduced forms of small proteins such as thioredoxin and glutaredoxin. Methionine sulfoxide, on its turn, is reduced back to methionine by the methionine sulfoxide reductases enzymes that are then recycled by the thioredoxin/thioredoxin reductase system.

**Figure 1:**
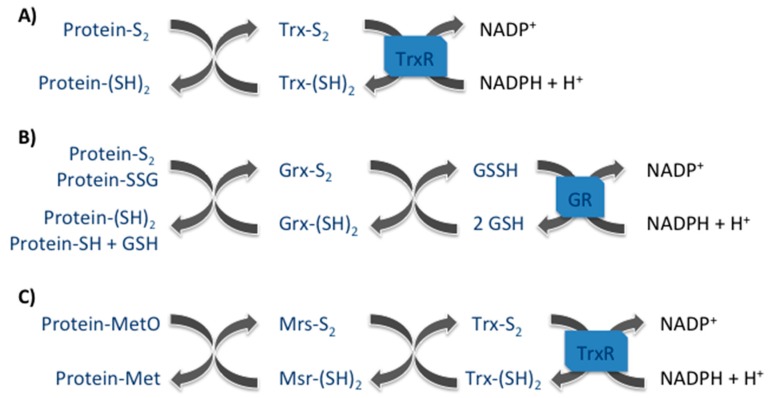
The three major protein repair systems. (**A**) Thioredoxin (Trx) system participates on the reduction of protein disulfide (Protein-S2); (**B**) Glutaredoxin (Grx) system reduces protein disulfide (Protein-S2) as well as protein glutathione mix disulfide (Protein-SSG); and (**C**) Methionine sulfoxide reductase (Msr) system reduces methionine sulfoxides (MetO).

Thioredoxins (Trx) are small ubiquitous proteins with two catalytic redox active cysteines (Cys-XX-Cys), which catalyze the reversible reduction of protein disulfide bonds. Subsequently, oxidized thioredoxins are reduced back enzymatically by the NADPH-dependent thioredoxin reductase (TR) enzymes, which together with NADPH and Trx constitute the thioredoxin system [1] (Figure 1). Two Trx enzymes have been identified to date, Txr1, which is present in the cytosol and can be translocated into the nucleus in oxidative stress conditions and Trx2, which is present in the mitochondria. The antioxidant activity of these enzymes consists of providing electrons to thiol-dependent peroxidases (Prx), allowing the recycling of these Prx enzymes for the continuous removal of reactive oxygen species (ROS) and reactive nitrogen species (RNS). Furthermore, Trx are also involved in the protection against protein oxidative damages by reducing methionine sulfoxide reductases (Msrs), enzymes capable of repairing oxidized methionines. In mammals, Trx also regulates the activity of many redox-sensitive transcription factors, such as NF-κB, Nrf2 and p53 [1].

Glutaredoxins (Grx) are found in almost all living organisms and collaborate with thioredoxins for the reduction of protein disulfides and S-glutathionylated proteins. Four Grx isoenzymes (Grx1, Grx2, Grx3, and Grx5) exist in mammals. In terms of structure, they belong to the Trx superfamily having a dithiol or monothiol active motive, Cys-XX-Cys or Cys-XX-Ser, respectively [2,3]. In contrast to Trx, Grx are reduced back non-enzymatically by glutathione, which is recovered by the glutathione reductase enzyme in the presence of NADPH (Figure 1). An exception was observed with the Grx2 isoenzyme, which was shown to be reduced by Trx2 in mitochondria [4]. The reduction of disulfides and the participation on protein deglutathionylation state the importance of Grx enzymes in defense against oxidative stress as well as in redox regulation of signal transduction [5,6].

Msrs in mammals constitute a group of four ubiquitous enzymes that catalyze the reduction of free and protein-derived methionine sulfoxides (MetO) to methionine (Met). Two diastereoisomers (rectus and sinister) of methionine sulfoxides can be formed upon protein methionine oxidation, methionine-R-sulfoxide (MetRO) and methionine-S-sulfoxide (MetSO). MsrA, which is present in the cytoplasm, in the nucleus and in the mitochondria reduces specifically the MetSO [7]. On the other hand, the three MsrB are responsible for the reduction of MetRO. In terms of their intracellular localization, MsrB1 is present in the cytoplasm as well as in nucleus, MsrB2 is only present in the mitochondria and MsrB3 is present in the mitochondria as well as in the endoplasmic reticulum of eukaryotic cells [7]. Msr enzymes possess one cysteine (a selenocysteine in the case of MsrB1), in their catalytic site, responsible for MetO reduction and whose recycling involves the formation of a disulfide bond with a second Msr cysteine. The disulfide bond can be further reduced restoring Msr activity by the Trx system (Figure 1). Msr have been intensely studied for their antioxidant roles as well as their protection against oxidative stress and apoptosis [8,9,10]. Furthermore, they have been suggested as being involved in longevity modulation of some models organisms such as *D. melanogaster* [11], *C. elegans* [12] and *S. cerevisiae* [13,14]. More recently, these enzymes have been considered as regulators of protein function [15] and as being involved in redox regulation of cellular signaling [16]. These proteins as well as their functions are further described and discussed in the following sections.

## 2. Methionine Sulfoxide Reductases Discovery

Due to the presence of a sulfur atom, methionine residues are very sensitive to oxidation leading to a modification or loss of protein function when oxidized within proteins. First evidences of the importance of keeping methionine in its reduced state for biological function appeared more than 70 years ago. Studying *L. arabinosus*, Waelsch and colleagues found that methionine oxidation of glutamine synthetase inhibited the conversion of glutamic acid into glutamine, an essential step for bacterial growth [17]. In addition, sporulation of *B. subtilis* was also described to be affected by methionine oxidation [18]. Few years after the identification of the first deleterious effects of MetO, a Msr activity, capable of reducing back MetO to Met was described, primarily in yeast [19], later in bacteria [20] and in higher organisms, such as plants [21] and animals [22]. Msr activity was evidenced in *E. coli* by their ability to grow in a culture medium with L-MetO as the only source of methionine, thus capable of catalyzing the reduction of MetO [20]. In 1981, Brot and colleagues partially purified one of the enzymes responsible for the reduction of MetO within proteins and showed it as essential for restoring the activity of the ribosomal protein L12 in *E. coli* [23]. The enzyme, later called MsrA, uses reduced Trx in vivo or dithiothreitol (DTT) in vitro as electron donor [24]. MsrA is a ubiquitous protein, differentially expressed in mammalian tissues and capable of reducing a variety of substrates such as free MetO and peptides or proteins containing MetO [25]. MsrA was found to be a stereospecific enzyme only capable of reducing the MetSO diastereoisomer of MetO, with an increased specificity for protein-bound MetO compared to free MetO [26,27].

Twenty years after the purification of MsrA, Grimaud et al. discovered that full reduction of oxidized calmodulin can be done by the combined action of MsrA and another enzyme called MsrB [28]. This new Msr is in fact responsible for the reduction of the MetRO diastereoisomer within proteins, which is not reduced by MsrA [29,30]. MsrB, later called MsrB1, SelX or SelR in mammals, was discovered in 1999 by Lescure and colleagues as a novel selenoprotein, but at this time, its function was unknown [31]. MsrB exclusively acts on peptidyl-MetO [28]. Intrigued by this, different authors have identified a novel Msr in *E. coli*, called fRMsr (for free MetRO reductase) or MsrC, which activity is specific for free MetO, being unable to reduce MetO present within proteins [30,32,33]. More recently, Gennaris and co-workers made another interesting discovery: they found a new Msr system, named MsrPQ (MsrP for periplasm and MsrQ for quinone), present in the envelope of *E. coli* bacteria, that, in contrast to the other known Msr, can reduce both MetO diastereoisomers using electrons directly from the respiratory chain, thus independently from Trx [34]. A similar system may exist in eukaryotic subcellular oxidizing compartments, such as the endoplasmic reticulum or lysosomes but, so far, fRMsr and MsrPQ systems were only found in prokaryotes or unicellular eukaryotes.

## 3. Methionine Sulfoxide Reductases Phylogenetic, Tissue and Cellular Distribution

The phylogenetic distribution of *Msr* genes was revealed by genomic analyses made in different organisms. These studies show the presence of *MsrA* and *MsrB* genes in all eukaryotes without exception. Bacteria can possess only *MsrA* genes, the two *Msr* genes or a bifunctional *MsrA/B* fusion gene [35]. This universal presence of *Msr* genes supports their essential roles for cell function, either in protecting them against oxidative damages as well as in regulating protein function. The greatest exception to the universal *Msr* representation among life domains is its absence in 12 archaea representative genomes [35]. Several hypothesis for this, although none of them was already been clearly proven, have been proposed: (i) the development of a functionally equivalent system; (ii) the low O_2_ solubility at high temperatures which would avoid ROS production within hyperthermophiles; (iii) the observation of non-enzymatic MetO reduction at higher temperatures [36]; (iv) the existence of protein structures that protect Met residues; (v) the presence of *Msr*-containing plasmids in these archaea; (vi) the discovery of fRMsr in some archaea including some of those lacking *Msr* genes [37] or finally (vii) the existence of an efficient first line of defense against ROS production (catalase, superoxide dismutases and Prx enzymes), which would create a low-ROS environment that diminishes the frequency of protein oxidation.

The fact that no *MsrB*-containing organism exists without *MsrA* gene suggests to the authors that these two genes evolved independently and that in these organisms, a greater abundance of the S epimer of MetO may explain that MsrA protein was sufficient to perform this specific protein repair process [35]. According to this hypothesis, MsrB may have evolved to play other redox functions, increasing defenses against greater oxidative damage seen in more complex life forms such as animals and plants. In addition, the organization of the two *Msr* genes also differs, this can explain the massive *MsrB* gene duplication seen in the plant *Arabidopsis thaliana*: nine *MsrB* genes in contrast to five *MsrA* genes [38]. In contrast to plants and algae [38], animals and bacteria contain fewer *Msr* genes: mammals have one *MsrA* and three *MsrB* genes while *E. coli*, *S. cerevisiae*, *C. elegans* and *D. melanogaster* have only one *MsrA* and one *MsrB* gene [29,39]. Such gene diversity underlines the biological importance of this system and gene redundancy may be explained by the necessity for organisms to respond to the modification of environmental conditions such as oxidative [10] or thermal stress [40,41].

In mammals, Msr enzymes are ubiquitously expressed [23,25], with the only exception of leukemic cells that do not express MsrA [42]. Analyses of mouse, rat, and human tissues revealed a maximal expression level of MsrA in kidney and liver, followed by heart, lung, brain, skeletal muscle, retina, testis, bone marrow, and blood [25,42]. The highest Msr activities were found in rat kidney [43] and human neutrophils [44], which in the case of neutrophils was later shown to be due mainly to MsrB type [45]. In human skin, MsrA was shown to participate to tissue homeostasis and to be a sensitive target for UV [46,47]. MsrA and all three MsrB proteins are expressed in melanocytes [48] and keratinocytes [46]. A lower MsrA expression was found in dermal fibroblasts [46] while a greater MsrA expression was found in sebaceous glands [49]. MsrB1 and MsrB3 were both expressed within vascular endothelial cells [49].

**Figure 2:**
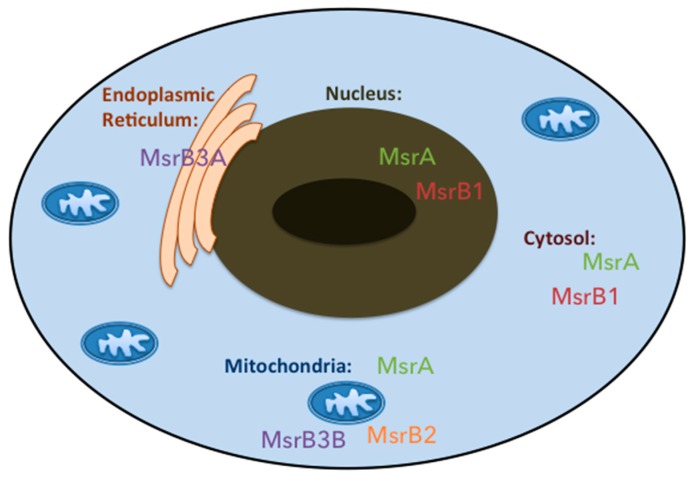
Subcellular distribution of methionine sulfoxide reductase enzymes in humans. MsrA and MsrB1 enzymes are present in the cytoplasm and in the nucleus of human cells. MsrA together with MsrB2 and MsrB3B enzymes can be found within mitochondria. The endoplasmic reticulum only contains a MsrB type enzyme called MsrB3A.

Msr enzymes are differentially distributed in the mammal subcellular compartments (Figure 2), which indicates that each Msr may have an organelle-specific role. MsrA is present in mitochondrial matrix due to its N-terminal mitochondrial signal sequence [50] but was also found in rat liver cytosolic fractions [51] and in the nucleus of mouse cells [52]. If its N-terminal peptide sequence was sufficient for mitochondrial targeting, other structural and functional elements present in the MsrA sequence can determine its intra-cellular distribution. Correctly folded MsrA is retained in the cytosol, while partially misfolded MsrA appears to be targeted to the mitochondria [52]. An alternative first exon splicing generating an additional MsrA form lacking a mitochondrial signal, which resides in cytosol and nucleus, was also evidenced [53]. This protein produced by initiation at the second site has been shown to be myristoylated and localized in the late endosomes [54].

For the MsrB family, the situation is more complex than for MsrA, due to the existence of four proteins resulting from the transcription and consequent translation of three different genes. The selenoprotein protein MsrB1 is present both in the nucleus and the cytosol, while MsrB2, also known as CBS-1, is present only in the mitochondria due to the presence of a N-terminal signal peptide [39]. Interestingly, MsrB3A and MsrB3B result from alternative splicing of the first exon of the *MsrB3* gene with MsrB3A displaying an endoplasmic reticulum signal peptide while MsrB3B showed a mitochondrial signal peptide at the N-terminus, in addition to an endoplasmic reticulum retention signal peptide at their C-terminus. In mouse, however, there is no evidence for *MsrB3* alternative splicing and the only MsrB3 protein is present in the endoplasmic reticulum, even though it has both the endoplasmic reticulum and the mitochondrial signal peptides at its N-terminus [55].

## 4. Methionine Sulfoxide Reductases Sequence, Structure and Catalytic Activity

**Figure 3:**
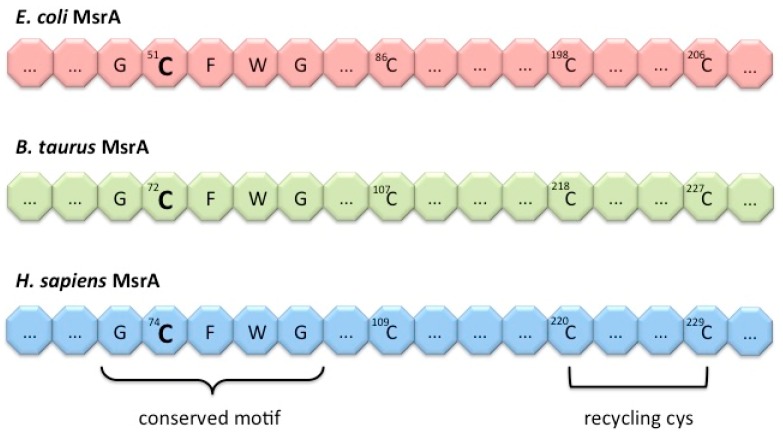
MsrA conserved GCFWG motif. Schematic representation of the conserved MsrA active-site sequence in *E. coli*, *B. Taurus* and *H. sapiens*, where the respective conserved cysteine is placed: Cys-51 in the case of bacterial MsrA, Cys-72 for bovine MsrA and Cys-74 in human MsrA. Three other cysteine residues were shown to be conserved: bacterial Cys-86, Cys-198 and Cys-206; bovine Cys-107, Cys-218 and Cys-227; and human Cys-109, Cys-220 and Cys-230; with the two last cysteines, toward the C-terminus of each MsrA, being responsible for recycling of the catalytic cysteine.

Sequence alignment between the primary structures of MsrA protein from different organisms showed that there is a high homology among them, with *E. coli* and *B. taurus* MsrA having 67% and 88% sequence identity to human MsrA [56]. In addition, this alignment highlighted a conserved active-site sequence GCFWG in all organisms studied. The strictly conserved cysteine within this motif (Cys-51 in the case of *E. coli* MsrA, Cys-72 in the case of bovine MsrA and Cys-74 in the case of human MsrA) is essential for the MsrA reducing activity (Figure 3). Moreover, three other cysteine were shown to be conserved within 70% of all MsrA proteins: Cys-86, Cys-198 and Cys-206 in the case of *E. coli*; Cys-107, Cys-218 and Cys-227 for bovine MsrA; and Cys-109, Cys-220 and Cys-230 in the case of human MsrA [56]. Toward the C-terminus, the two last cysteines bracket a glycine-rich region on MsrA sequence and may serve as additional recycling cysteine for the catalytic mechanism.

MsrA three-dimensional (3D)-structures obtained by X-ray crystallography from *E. coli* [57], *B. Taurus* [58], *M. tuberculosis* [59] and *P. trichocarpa* [60] were also essential to determine the mechanistic aspects of MsrA catalysis. MsrA folding belongs to an α/β class of proteins. The presence of the catalytic cysteine in the N-terminal α-helix of the protein allows it to face MetO residues present in other proteins, while the two recycling cysteines are buried in the C-terminal region of the protein. Analysis of the 3D structures of the MsrA with MetO showed that the oxygen of the methionine sulfoxide is strongly stabilized by a hydrophilic subsite composed of a network of hydrogen bonding interactions including Tyr-82, Glu-94 and Tyr-134 in MsrA (numbers based on the *E. coli* MsrA sequence) [61]. Using NMR technology, a high degree of flexibility of the C-terminal region of oxidized MsrA was evidenced, which favors the formation of an intramolecular disulfide bond between the two recycling cysteines [62].

**Figure 4:**
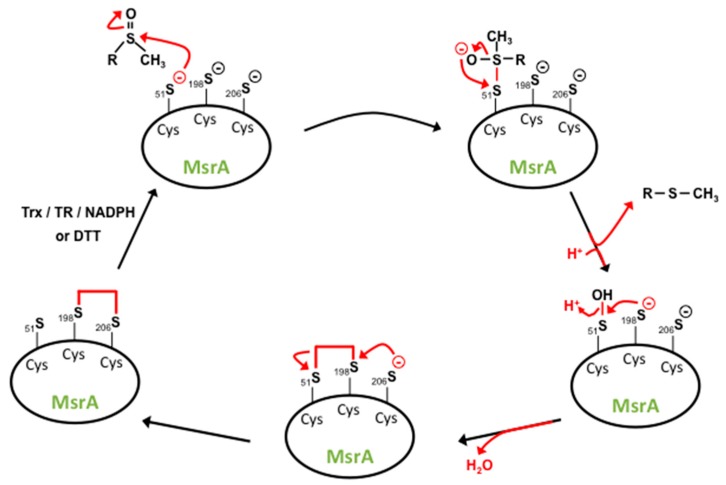
MsrA catalytic mechanism. The nucleophilic attack of the MsrA catalytic cystein (Cys)-51 on the sulfur atom of the methionine sulfoxide substrate leads to the formation of an unstable intermediate (enzyme bound to the substrate). Bacterial MsrA is used in this representation. Ionic rearrangement leads to the formation of a sulfenate ion with the concomitant release of the methionine molecule and protonation of the sulfenate ion to produce a sulfenic acid intermediate on MsrA. The nucleophilic attack of the recycling Cys-198 on the sulfur atom of the sulfenic acid intermediate leads to the formation of an intramolecular disulfide bond. MsrA full native state recovery is achieved after another nucleophilic attack of Cys-198 on Cys-206 generating a second intramolecular disulfide bond, which can be reduced either by the thioredoxin (Trx)/thioredoxin reductase (TR)/NADPH regenerating system or by dithiothreitol (DTT) [60,63].

The catalytic mechanism for MsrA has been described by Boschi-Muller and colleagues in the case of *E. coli* MsrA and by Lowther et al. for bovine MsrA [58,63]. Based on sulfenic acid chemistry, these two groups proposed a reaction mechanism for MsrA catalysis consisting in several steps (Figure 4). First, the catalytic Cys-51/72 will act as a nucleophilic agent attacking the sulfoxide moiety of the substrate leading to formation of a sulfenic acid on the catalytic cysteine with the concomitant release of 1 mol of methionine per mol of Msr. Subsequently, the recycling Cys-198/218 attack on the sulfenic intermediate will create an intramolecular disulfide bond between the catalytic and the recycling cysteine. In the case of another recycling cysteine, such as Cys-206 from *E. coli* and Cys-227 from *B. taurus*, there is subsequent nucleophilic attack of Cys-206/227 on Cys-198/218 creating a new intramolecular disulfide bond between these two recycling cysteines. The last step involves reduction of the disulfide bond by Trx in vivo or other reducing agents, such as DTT in vitro. Kinetic studies showed that the rate of formation of the sulfenic acid is high while the recycling process, which reduces back the oxidized catalytic cysteine, is overall rate-limiting [64]. Murine and human MsrA possess the same mechanism of catalysis but in the case of other bacteria, such as *N. meningitides* [65] and *M. tuberculosis* [59], MsrA proteins possess only one recycling cysteine equivalent to Cys-198 from *E. coli*. Recently, MsrA has been shown to also have an oxidase activity towards methionines, producing MetSO within proteins, including itself, or on free methionines [66], even in the presence of Trx [67].

The first protein showing a methionine-R-sulfoxide reductase activity was identified in *E. coli* and was named MsrB. It has no similarity with MsrA, presents 43% sequence homology with PilB from *N. gonorrhoeae* and contains a conserved signature sequence CGWP(S/A)F [28]. Indeed, the PilB protein has a MsrA- and a MsrB-like C-terminal domains that function with opposite substrate stereospecificity and a N-terminal thioredoxin-like domain that allows the regeneration of both Msr active sites [30,68,69]. The 3D-structures from the MsrB domain of PilB obtained by X-ray crystallography showed no similarity with the one from MsrA [68]. However, the active sites for both enzymes show an axial symmetry as if they were reflecting each other in a mirror, which can be explained by the stereospecificity of the two enzymes. This symmetry suggests a similar catalytic mechanism for both enzymes and indeed, the two catalytic cysteines of PilB, Cys-495 and Cys-440, function in a similar way to the *E. coli* MsrA Cys-51 and Cys-206: a nucleophilic attack by Cys-495 to MetO leads to the production of a trigonal intermediary compound that after ionic rearrangement and subsequent methionine release, will form a sulfenic acid on PilB Cys-495 [68]. As for MsrA, a series of proton exchanging events occurs leading to the formation of a disulfide intramolecular bond, which is consequently reduced by Trx [68].

MsrB1 is a mammalian MsrB enzyme that shows less homology with MsrBs from invertebrates, with only 29% of similarity between the sequences of human MsrB1 and the MsrB domain of *N. gonorrhoeae* PilB [31]. The presence of the selenium atom in its active site is critical for the catalytic function of this enzyme. In fact, the wild type selenoprotein MsrB1 is 800-fold more active than the corresponding cysteine-MsrB1 mutant form [39]. Similarly, a cysteine to selenocystein mutation in mammalian MsrB2 and MsrB3 resulted in 100-fold increase in the catalytic activity of the enzymes [70]. The incorporation of a selenocysteine into the primary sequence of MsrB1 protein is due to a SECIS (SelenoCysteine Insertion Sequence) element localized in the 3′UTR of MsrB1 mRNA at a distance from the UGA codon [31]. The presence of a recycling cysteine, present in the N-terminal region of the MsrB1 protein is important for resolving the selenic acid intermediate formed during catalysis [70].

MsrB2 was first identified in humans as a 21 kDa protein composed of 202 amino acids and carrying the conserved MsrB sequence GTGWP [71]. It presents 59% homology with *E. coli* MsrB and 42% with the C-terminal domain of *N. gonorrhoeae* PilB. MsrB2 is four times less efficient than MsrA for the reduction of a specific synthetic substrate. Similarly to MsrB1, MsrB2 possesses a CXXC motif responsible for zinc binding. In contrary to other MsrB, the mammalian MsrB2 proteins do not contain a recycling cysteine residue in the middle of the sequences and the sulfenic intermediate could be directly reduced by Trx [70].

**Figure 5:**
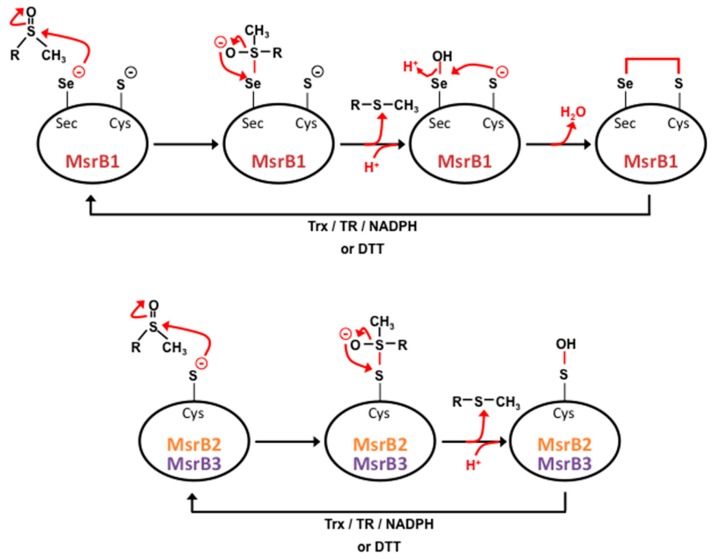
MsrB catalytic mechanism. In the case of MsrB1 (upper part of the figure), methionine sulfoxide reduction starts with the nucleophilic attack of selenocystein (Sec) on the sulfur atom of the substrate leading to the formation of an unstable intermediate. Ionic rearrangement leads to the formation of a selenic acid intermediate with the concomitant release of a methionine molecule. The nucleophilic attack of the recycling cysteine (Cys) on this selenic acid intermediate leads to the formation of an intramolecular selenenylsulfide bond, which is subsequently reduced by the thioredoxin (Trx)/thioredoxin reductase (TR)/NADPH regenerating system or by dithiothreitol (DTT). In contrast, the sulfenic intermediate of MsrB2 and MsrB3 (lower part of the figure), formed on Cys after methionine release, can be directly reduced to its fully active state by one of these mechanisms [68,70].

Another gene, that seems to be only present in mammals’ genome, encodes, by alternative splicing, two proteins of the MsrB family, MsrB3A and MsrB3B [39]. As for the other MsrB proteins, they contain a catalytic cysteine and the CXXC motif responsible for zinc binding. In terms of their catalytic activity, these enzymes act similarly to the MsrB2 enzymes (Figure 5). Despite of the presence of cysteine residues instead of selenocystein in their active sites, MsrB2 and MsrB3 exhibit good catalytic efficiencies but that are slightly lower than MsrB1 [39,70].

First studies on the biological reducing agents for MsrA revealed that reduced Trx, high levels of DTT or reduced lipoic acid can reduce oxidized MsrA in vitro. If *E. coli* MsrA and MsrB and bovine MsrA efficiently use either Trx or DTT as reducing agents, human MsrB2 and MsrB3 showed less than 10% of their activity with Trx as reducing agent when compared to the use of DTT [72]. This suggests that in animal cells, Trx may not be the only reducing power for these two enzymes. Thionein, the reduced metal-free form of metallothionein, and selenium compounds, such as selenocystamine, could also function as reducing agents for human MsrB3 and MsrB2 [72,73]. Furthermore, it was found that a few Msr such as *A. thaliana* MsrB1, *Clostridium* Sec-containing MsrA or the red alga *G. gracilis* MsrA can be regenerated by Grx/glutathione system [74,75,76,77]. In the particular case of *A. thaliana* MsrB1, the sulfenic acid is reduced by glutathione forming a glutathionylated intermediate that is attacked by glutaredoxins [76]. However, the rate of the recycling process by the Grx system is at least 10 to 100-fold lower compared to Trx acting in Msr with a recycling cysteine, suggesting that the Grx system would not be used in Msr in which a disulfide bond is formed. In agreement with this hypothesis is the fact that methionine auxotrophic *E. coli* is unable to grow in the presence of MetO when the *Trx1* gene is inactivated [78].

## 5. Methionine Sulfoxide Reductases in Protection Against Oxidative Stress

Several studies using different bacterial and eukaryotic models revealed that Msr enzymes and methionine amino acids work together in cellular protection against oxidative stress. Surface-exposed methionine residues in proteins are more easily oxidized by ROS, such as H_2_O_2_, chloramines or HOCl, due to the presence of sulfur atoms. However, they are believed to be more resistant to oxidative inactivation [79], thus keeping protein structure and catalytic function. Through this mechanism, methionines are proposed to act as a threshold barrier for other amino acid oxidation, which would lead to loss of the protein activity [79,80]. Another mechanism through which methionine can act as antioxidant amino acid, observed in *E. coli* [81], *S. cerevisiae* [82] and in mammalian cells [83], is its misacylation, i.e., the incorporation of methionines by non-methionyl-tRNAs during translation. To date, this translation infidelity was revealed to be specific to methionines and its frequency of occurrence was shown to increase upon innate immune or chemically induced oxidative stress [83], thus suggesting it plays a role in protecting against these kinds of stresses. In agreement, some Met-mistranslated forms were observed in the catalytic domain of the Ca^2+^/calmodulin-dependent kinase II (CaMKII) under Ca^2+^ stress, resulting in increased catalytic activity as well as alterations of proteins subcellular localization [84].

The antioxidant protection conferred by methionine residues is also due to their cyclic reduction by Msr enzymes, which by rendering methionines prone to new oxidation reactions, will lower ROS levels [85]. In fact, the absence of MsrA expression leads to reduced *E. coli* as well as *M. tuberculosis* viability when treated with H_2_O_2_, nitrite or S-nitrosoglutathione; effect that can be reversed by transformation of the mutant strain with a plasmid containing the wild-type MsrA gene from the respective bacteria species [86,87]. This MsrA protection against an oxidative stress state induced by different oxidants has been also verified in other bacteria such as *O. anthropi* [88] and *S. aureus* [89]. Moreover, the presence of MsrA was relevant for survival of *M. smegmatis* within macrophages producing high levels of ROS and RNS as a defensive response against those microorganisms [90]. Growth alteration and significant protein carbonyl accumulation were also observed in MsrA null yeast mutants when submitted to H_2_O_2_ treatment [91,92]. In agreement, overexpression of MsrA in this eukaryotic species reduced the levels of free and protein-bound MetO leading to increased resistance to toxic concentrations of H_2_O_2_ [93]. Overall the studies indicate that bacteria and yeast are dependent of MsrA to counteract the damaging effects of oxidative stress and consequently for their survival in these conditions. In the case of *S. cerevisiae* not only MsrA, but also MsrB was shown to protect against oxidative damages mediated by the toxic metal chromium [94].

Msr protection against oxidative stress is also notorious in higher organisms such as plants and animals. During the dark periods when *A. thaliana* produces more H_2_O_2_, it has been shown that the Msr system was important to prevent protein oxidative damage, thus minimizing protein turnover in these conditions of limited energy supply [95]. MsrB3 was identified in a proteomic study as a cold-responsive protein in Arabidopsis [96] as essential to reduce oxidized methionine and to lower H_2_O_2_ level that accumulates in the endoplasmic reticulum during plant cold acclimation [40]. In the case of invertebrate animals, overexpression of MsrA in the nervous system of *D. melanogaster* was shown to increase the resistance of these transgenic animals to paraquat-induced oxidative stress [97], while the suppression of this gene in *C. elegans* resulted in worms more sensitive to paraquat treatment, presenting chemotaxis and locomotor failure, partly due to muscle defects [12]. In line with these results, the induction of MsrA by ecdysone, was also shown to protect *Drosophila* against H_2_O_2_-induced oxidative stress [98].

In mammals, paraquat injection decreased the survival of *MsrA^−/−^* mice comparing to *MsrA^+/+^* or *MsrA^+/−^* mice [9]. MsrA null mutant mice present also higher level of protein carbonyls in the liver, kidney [99] and heart, correlated with mitochondria morphological changes in this last tissue [100]. Regarding the heart, our laboratory observed that the modulation of Msr activity in rat hearts along the course of cardiac ischemia/reperfusion may involve structural modification of the enzyme rather than a modification of MsrA protein level [101]. Indeed, later it was shown that the MsrA cytosolic form needs to be myristoylated in order to confer heart protection against ischemia/reperfusion damages, suggesting that it must interact with a hydrophobic domain [102]. This protective role of MsrA against ischemia/reperfusion injuries was also evidenced in mouse kidney, with increased oxidative stress markers, inflammation and fibrosis observed in kidneys of *MsrA^−/−^* mice after injury comparing to wild type [103,104]. In agreement to what have been found in *MsrA^−/−^* mice, increased GSH, protein and lipid oxidation were also found in the liver and kidney of the *MsrB1* K.O. mice [105]. MsrB1 is a selenoprotein, thus depending on selenium concentrations to be produced. Interestingly, Jacob Moskovitz has found that *MsrA* K.O. mice submitted to a selenium deficient diet presented decreased MsrB activity but also less Gpx and Trx activities in their brains [106]. MsrA or MsrB1 deficiency significantly accelerates acetaminophen-induced hepatic toxicity by aggravating GSH depletion and lipid peroxidation [107].

The protective role of Msr enzymes against oxidative damages was also suggested by various studies in different mammalian cell types in vitro, such as retinal pigment epithelial cells from human [108], monkey [109], rat [110], or human lens cells [111]. Overexpression of *MsrA* in human lens cells, for instance, gave them an increased resistance to H_2_O_2_-induced stress while *MsrA* gene silencing led to an their increased sensitivity towards oxidative treatment and to a loss of viability even in the absence of exogenously added stress [111]. On the other hand, silencing of all or individual *Msr* genes led to increased oxidative stress-induced cell death indicating that MsrB are also implicated in human lens epithelial cell viability in these conditions [112]. MsrB1 was the most studied MsrB in human lens epithelial cells. Silencing of this *Msr* gene resulted in increased ROS levels, lipid oxidation, ER stress, decreased mitochondrial potential and release of cytochrome c, ultimately leading to caspase-dependent apoptosis [113,114,115]. Furthermore, peroxynitrite treatment to MsrB1-deficient human lens epithelial cells will aggravate the oxidative damages and F-actin disruption, that normally occurs after this nitric stress [116], suggesting that MsrB1 protects lens cells from F-actin nitration. Using stable human embryonic kidney HEK293 clones with an altered Msr system due to silencing the expression of *MsrA*, *MsrB1*, or *MsrB2*, our laboratory performed a proteomic analysis on the Msr-silenced cells grown under basal conditions or submitted to oxidative stress, revealing that the disruption of the Msr system mainly affects proteins with redox, cytoskeletal or protein synthesis, and maintenance roles [117]. Interestingly, most of the proteins found altered in the Msr mutants were also identified as potential Msr substrates and have been associated with redox or ageing processes in previous studies. Furthermore, we and others have shown that human T lymphocyte cells presented an increase resistance to H_2_O_2_ or zinc treatments when transfected with *MsrA* and/or *MsrB2* genes by reducing the levels of intracellular ROS species and protein oxidative damages that would lead to cell death [8,93,118]. The role for MsrA in the prevention against the accumulation of protein and cellular oxidative damage provoked by H_2_O_2_-induced oxidative stress was also studied in fibroblast cells [119] and was associated in these cells to MsrA-dependent differentially expression proteins implicated in protection against oxidative stress, apoptosis, and premature ageing [120]. Even though overexpression of MsrA in the endoplasmic reticulum of mammalian cells increases their resistance to oxidative and endoplasmic reticulum stresses [121], the resistance to an endoplasmic reticulum stress is mainly conferred by MsrB3 [41,122]. Finally, in human skin cells, the behavior of the MsrA enzyme seems to be dependent on the type of ultraviolet (UV) exposure and the dose applied, suggesting a hormetic response to environmental stress. In fact, low doses of UVA stimulate MsrA expression, while UVB or high doses of UVA contribute to decrease MsrA expression and increase protein carbonyl [46,47], a profile that can be prevented by pre-treating the cells with MsrA [123]. In melanocytes, the absence of MsrA expression also increased sensibility to oxidative stresses and cell death even in the absence of exogenous stresses [124].

Together, these studies suggest that methionine amino acid residues along with the Msr system constitute a potent antioxidant ROS scavenging system, preserving macromolecules in their reduced state and thus contributing to protein homeostasis and function while protecting different cell types and organisms from different kinds of oxidative stresses.

## 6. Methionine Sulfoxide Reductases in Disease, Ageing and Longevity

Given that Msr system also protects proteins from irreversible oxidation as a result of a severe oxidative stress and that protein carbonyls levels are usually referred as a marker of oxidative stress in pathophysiological conditions and during ageing, it is expected that Msr would be implicated in diseases and in ageing process.

While it has been shown that oxidized proteins accumulate in tissues from patients exhibiting age-related diseases such as Alzheimer’s, Parkinson’s, and Huntington’s diseases, and cataracts [125,126], reduced MsrA activity was found in the brains of Alzheimer’s disease patients [127]. In agreement with this is the fact that *MsrA* K.O. mice demonstrated behavioral abnormality (tip-toe walking) consistent with cerebellar dysfunction [99], increased light scattering—a common cataract symptom [128] and enhanced neurodegeneration with characteristic features of neurodegenerative diseases [129]. Methionine oxidation in Met-35 of amyloid ß-peptide (Aβ peptide) is thought to be critical for aggregation and neurotoxicity [130] and it was shown that the absence of MsrA modifies Aβ solubility properties and causes mitochondrial dysfunction in a mouse model of Alzheimer’s disease [131,132]. In the case of Parkinson’s disease, oxidation of the methionine residues in α-synuclein is thought to be the main reason of protein fibrillation causing the pathology [133].

The importance of Msr in age-related diseases and the accumulation of modified proteins produced by the action of ROS as major hallmark of ageing, suggests that Msr would have also an important role in ageing phenotype. Indeed, the accumulation of oxidatively modified proteins during ageing has been largely attributed to declined efficacy of the systems involved in protein homeostasis such as protein degradation and protein repair [134]. Again, our laboratory has shown that MsrA is down-regulated in aged rats [135] and during replicative senescence of fibroblasts [136]. Both cytosolic and mitochondrial Msr activities were found to decline upon replicative senescence [137] and increased MetO levels were found in membrane proteins of senescent erythrocytes [138] as well as in senescent *E. coli* [139].

Several studies have been done to elucidate the implication of the Msr system in regulating lifespan but they are still controversial. The first two groups having tried to test this hypothesis used two different models: *MsrA* K.O. mice and *MsrA* overexpressing *Drosophila* [97,99]. Knockout of the *MsrA* gene in mice reduced its lifespan by 40% [99] while, in contrast, its overexpression in *Drosophila* accounted for a 70% extension in their healthy lifespan [97]. In both studies, MsrA-dependent lifespan modulation was related to its role in protection against oxidative stress but later, another study showed that while the lack of MsrA in mice increases sensitivity to oxidative stress, it does not diminish lifespan [9]. While the discussion about the effects of Msr system on the late survival of higher animals is still open, MsrA overexpression, however, was shown to increase lifespan of *S. cerevisiae* [13] whereas its inhibition in yeast or in *C. elegans* is accompanied by a shorten lifespan [12]. Moreover, ectopically expression of fRMsr in fruit flies, an enzyme lost during evolution, that reduces free MetO, increases stress resistance and extends lifespan of animals [140].

Until now, overall these studies agreed that the importance of Msr system in ageing and neurodegenerative diseases was dependent of its role as antioxidant enzyme protecting cells and organisms from the deleterious effects of oxidative stress. The discovery that alternation between methionine oxidation and reduction could serve as regulator of protein function, as reviewed below, raises the hypothesis that the Msr role on ageing and survival could also come from these intracellular signalling functions.

## 7. Methionine Sulfoxide Reductases as Regulators of Protein and Cellular Functions

Evidences that the cyclic interconversion between Met and MetO within proteins is implicated in the regulation of cell signaling functions are gaining space in the field of Msr studies. In general, it has been demonstrated that the oxidation of certain methionine residues in proteins induces mainly the loss of their biological activity, while their reduction by Msr is capable of reversing it. The types of proteins in which methionine oxidation has been involved in their function are very diverse: proteases or protease inhibitors, metabolic enzymes, cytoskeleton proteins, cytokines, heat shock proteins, hormones, heme proteins, proteins associated with neurodegenerative disorders, proteins involved in immunodefences, as well as different bacterial proteins and snake toxins (see [141] for review). Among the more than 50 proteins reported to have altered activity due to formation of MetO, only part of them was already described as being substrates of Msr enzymes either in vitro or in vivo experiments. In this last section, we will address some examples of these Msr substrates.

The *E. coli* ribosomal protein L12 was the first characterized substrate of MsrA [23]. Oxidation of three of its methionines by H_2_O_2_ decreases its ability to bind to ribosomes and to interact with other ribosomal proteins such as L10, impairing protein synthesis [142], while MsrA reduction of the oxidized methionines abled L12 ribosomal protein to regain its activity [23]. Another *E. coli* protein involved in protein machinery is the Ffh component of the ubiquitous signal recognition particle. This protein contains a methionine-rich domain whose oxidation compromises Ffh interaction with a small RNA. Oxidized Ffh is a substrate for MsrA and MsrB enzymes and reduction of Ffh MetO residues allows the recovery of its RNA-binding abilities [143].

Regarding serine protease inhibitors, methionine oxidation was also associated with a loss of function in α1-antitrypsin [144] and α2-antiplasmin [145]. In particular, α1-antitrypsin protein is important for lungs protection by preserving anti-neutrophil elastase activity, which is associated with the risk of developing emphysema. Oxidation of two methionines of α1-antitrypsin causes a loss of the anti-neutrophil elastase activity, which can be restored in part with the addition of MsrA in vitro [146]. Moreover, methionine oxidation of Human Immunodeficiency Virus 2 (HIV-2) protease, which cleaves proteins involved in the HIV-2 reproductive cycle, also inhibits its proteolytic activity while the addition of only MsrA partially restores it [147]. Another important protein participating in the immunological and oxidative stress response, the inhibitor of kappa B-alpha (IκBα), named for its inhibition activity of the transcription factor nuclear factor-κB (NF-κB), can also be oxidized on a methionine residue thereby increasing its resistance to proteasomal degradation [148]. When IκBα is oxidized by taurine chloride or chloramines, it cannot dissociate from NF-κB, thus preventing it from nucleus translocation and subsequent activation of its target genes [149]. Inhibition of NF-κB activation is prevented by MsrA [150]. Together, these findings could lead to new therapeutic strategies in order to fight against diseases such as pulmonary emphysema, AIDS or immunological diseases where NF-κB play an important role.

Methionine oxidation and its Msr-dependent reduction was also shown to be important for the regulation of cellular excitability, in particularly through the regulation of the voltage-gated and the calcium (Ca^2+^)-activated potassium channels [151,152]. Another protein involved in cellular excitability, and whose activity is regulated by Msr enzymes, is the Ca^2+^-binding protein calmodulin (CaM) [153]. This protein detects the calcium signals in cells and coordinates energy metabolism, which in turn produce superoxide in the mitochondria. CaM loses its conformational stability upon Met oxidation [154] thus failing to activate the plasma membrane Ca^2+^-ATPase [155]. In contrast, its full reduction by MsrA and MsrB leads to its binding to the inhibitory domain of the plasma membrane Ca^2+^-ATPase, inducing helix formation within the CaM-binding sequence and releasing enzyme inhibition [28,156]. Full reduction of CaM by MsrA and MsrB was also found to restore its binding to *B. pertussis* adenylate cyclase [157]. Thus, one can think that upon oxidative stress, increasing levels of cytosolic Ca^2+^ are probably the consequence of the oxidation of specific methionines in CaM that is no longer able to activate the Ca^2+^-ATPase in the plasma membrane. As a consequence, accumulation oxidized CaM will result in down-regulation of cellular metabolism thus, controlling the generation of ROS.

One of the CaM proteins targets, the Ca^2+^/calmodulin-dependent protein kinase II (CaMKII), is also regulated by methionine oxidation. Indeed, apart from Ca^2+^/CaM regulation or its autophosphorylation at Thr-287, oxidation of one of its methionine residues leads also to its Ca^2+^/CaM-independent activity [158]. This is consistent with the notion that Met oxidation does not invariably induce enzyme inactivation. The same authors have shown that MsrA enzymes are essential for reversing CaMKII oxidation in myocardium in vivo [158] and this oxidation of CaMKII mediates the cardiotoxic effects of aldosterone, while MsrA overexpression reversed its effects [159].

If Met oxidations are involved in the regulation of biological functions, we could think that they cannot depend only on random oxidation by multiple forms of ROS. There should be also specialized oxidases that catalyze the oxidation of specific protein targets. Indeed, Hung and colleagues have found that a flavoprotein monooxidase (FMO) called Mical is able to bind to F-actin and to selectively oxidized 2 of its 16 methionine residues into the R stereoisomer of MetO, resulting on actin disassembly in vitro and in vivo [160]. This Mical-dependent redox regulation of actin that can be reversed by MsrB1/SelR, was involved in bristles formation in *Drosophila* [161], in membrane trafficking in bone marrow-derived macrophages [15] and in lens epithelial cells [116]. In addition to the Mical enzyme, other mammalian FMO mainly involved in oxidative xenobiotic metabolism in liver and kidney, have been shown to exhibit methionine oxidase catalytic activity. In *Aspergillus nidulans*, FMO-mediated oxidation of a specific methionine residue regulates the subcellular localisation of the transcription factor NirA [162].

The increasing work done in the reversibility of methionine oxidation in vitro as well as in vivo led to the accepted view that this sophisticated redox based mechanism must be considered as other post-translational modifications on proteins such as phosphorylation, acetylation or glutathionylation, implicated in the regulation of many important protein and cellular functions.

## 8. Conclusions

In this paper, we have tentatively provided a comprehensive review of the Msr system in terms of its discovery and biologically relevant characteristics as its important physiological roles in protecting against oxidative stress, in ageing and in regulating protein function. Indeed, since the specific reduction of L-methionine sulfoxide has been evidenced and the first Msr, now referred to as MsrA, partially purified and characterized in 1981 [23], much progress has been achieved in terms of both MsrA and MsrB, discovered 20 years later [28], gene organization and subcellular localization in different species from bacteria to mammals, as well as their enzymatic and structural characterization. Interestingly, the Msrs system is also connected to selenocysteine biology since mammalian MsrB1 is a selenoprotein [39].

Beside restoration of protein structure and function through the selective reduction of MetO within the polypeptide chain, protection against oxidative stress has been shown to also result from the cyclic reduction of protein surface exposed MetO by Msr enzymes, acting as a protein built-in antioxidant system [163].

Hence, methionine amino acid residues together with the Msr system constitute a potent ROS scavenging system, preserving proteins in their reduced state and thus protecting different cell types and organisms from oxidative stress, ultimately contributing to protein homeostasis and cell survival in endogenous or exogenous stress conditions.

Since accumulation of oxidized proteins is one of the hallmarks of ageing and many age-associated diseases, the fate of the Msr system has been investigated in these situations. The implication of the Msr system in regulating lifespan of model organisms has been studied with either overexpressing or knock-out strains for either *MsrA* and/or *MsrB* leading at first sight to somehow contradictory results that may be due to the multifunctionality of the Msr system and the complex relationship between longevity, protection against oxidative stress and redox regulation of signaling pathways.

Another important topic for which the Msr system has been implicated is the regulation of protein function and hence cellular signaling. Methionine oxidation of the first identified protein targets was mainly associated with a loss of function that was restored by the Msr system. However, the activation of the CaMKII by methionine oxidation in the absence of Ca^2+^/CaM [158] has opened up the possibility that the Msr system could also play an important role in the regulation of protein function. The role of selective methionine oxidation of actin by monooxygenases that results in its disassembly which is reversed by MsrB1 [162] have further strengthened the concept that methionine oxidation and the Msr system indeed play a critical role in regulation protein function. Although, a number of protein targets for methionine oxidation and its reversion by the Msr system has already been identified, identification of new targets still needs to be achieved to appreciate the physiological relevance of this sophisticated redox based cellular regulatory system. Unfortunately, attempts to raise specific antibodies aimed at detecting MetO in proteins, which would be of great value for identifying protein targets, have been unsuccessful so far [164]. However, new alternative methods such as these aimed at characterizing in vivo methionine oxidation using mass spectrometry based and proteomics methods [165], and those aimed at determining the concentration of protein based MetO using fluorescent biosensor technology [166] are expected to be of valuable interest for further investigating the Msrs system and its role in redox biology.
